# Development and Evaluation of a Serious Game Application to Engage University Students in Critical Thinking About Health Claims: Mixed Methods Study

**DOI:** 10.2196/44831

**Published:** 2023-05-11

**Authors:** Ida-Kristin Orjasaeter Elvsaas, Lisa Garnweidner-Holme, Laurence Habib, Marianne Molin

**Affiliations:** 1 Department of Nursing and Health Promotion Faculty of Health Sciences Oslo Metropolitan University Oslo Norway; 2 Department of Computer Science Faculty of Technology, Art and Design Oslo Metropolitan University Oslo Norway; 3 Department of Health and Exercise Kristiania University College Oslo Norway

**Keywords:** game application, critical thinking, critical health literacy, Informed Health Choices, evaluation, mixed methods study, serious games, behind the headlines, mobile phone

## Abstract

**Background:**

Misleading health claims are widespread in the media, and making choices based on such claims can negatively affect health. Thus, developing effective learning resources to enable people to think critically about health claims is of great value. Serious games can become an effective learning resource in this respect, as they can affect motivation and learning.

**Objective:**

This study aims to document how user insights and input can inform the concept and development of a serious game application in critical thinking about health claims in addition to gathering user experiences with the game application.

**Methods:**

This was a mixed methods study in 4 successive phases with both qualitative and quantitative data collected in the period from 2020-2022. Qualitative data on design and development were obtained from 4 unrecorded discussions, and qualitative evaluation data were obtained from 1 recorded focus group interview and 3 open-ended questions in the game application. The quantitative data originate from user statistics. The qualitative data were analyzed thematically, and user data were analyzed using nonparametric tests.

**Results:**

The first unrecorded discussion revealed that the students’ (3 participants’) assessment of whether a claim was reliable or not was limited to performing Google searches when faced with an ad for a health intervention. On the basis of the acquired knowledge of the target group, the game’s prerequisites, and the technical possibilities, a pilot of the game was created and reviewed question by question in 3 unrecorded discussions (6 participants). After adjustments, the game was advertised at the Oslo Metropolitan University, and 193 students tested the game. A correlation (*r*=0.77; *P*<.001) was found between the number of replays and total points achieved in the game. There was no demonstrable difference (*P*=.07) between the total scores of students from different faculties. Overall, 36.3% (70/193) of the students answered the evaluation questions in the game. They used words such as “fun” and “educational” about the experiences with the game, and words such as “motivating” and “engaging” related to the learning experience. The design was described as “varied” and “user-friendly.” Suggested improvements include adding references, more games and modules, more difficult questions, and an introductory text explaining the game. The results from the focus group interview (4 participants) corresponded to a large extent with the results of the open-ended questions in the game.

**Conclusions:**

We found that user insights and inputs can be successfully used in the concept and development of a serious game that aims to engage students to think critically about health claims. The mixed methods evaluation revealed that the users experienced the game as educational and fun. Future research may focus on assessing the effect of the serious game on learning outcomes and health choices in randomized trials.

## Introduction

### Background

Unreliable claims about health and treatment effects are common in the media [1,2], and many lack the ability to critically assess these claims [3,4]. The inability to critically assess health claims can lead to poor health choices including the overuse of unnecessary treatments [5] and underuse of effective treatments [6], which can negatively affect individual health and health systems. Recently, the flow of unreliable and incorrect information about the COVID-19 pandemic caused confusion and risk-taking behavior that could harm health, and the World Health Organization called this information overload an “infodemic” [7] that calls for action [8].

Because of the potential negative consequences of acting on unreliable claims, there is great value in developing effective learning resources to enable people to think critically about health claims and make informed choices [9]. In this regard, digital games have become increasingly popular and have become an integral part of educational methods, including training professional behaviors and attitudes [10]. A scoping review of serious games for health professional education revealed that the use of serious games improves short-term learning and provides an increased sense of engagement and immersion among users [11]. Most commonly, this was credited to the games' competitive element of fun and interactivity. In particular, serious games installed on smartphones or other mobile learning platforms seem to trigger positive attitudes such as enhanced interest and motivation to learn [10]. A recent systematic review mapping the theoretical underpinning approach used in gamification, serious games, and game-based learning identified self-determination theory as the most widely used theory [12]. Using self-determination theory, addressing intrinsic motivation to learn, as opposed to extrinsic motivation, is viewed as essential to improve learning outcomes [13]. In addition, serious games can also be effective in facilitating a holistic understanding of scientific concepts [10].

### A Serious Game for Critical Thinking About Health Claims

#### Overview

In previous work, we have described how unreliable health claims in the media such as news stories, social media posts, and advertisements, can be used to teach students how to critically assess health claims [14]. Given the potential of using serious games to trigger and motivate students to learn more about a topic, we wanted to explore the possibilities for expanding our work to include a serious game to engage university students to think critically about health claims in the media as part of an umbrella project called “Behind the headlines” [15].

The prerequisite for the game was to address the same key concepts for assessing health claims that were addressed in “Behind the headlines” [14]. “Behind the headlines” included 5 key concepts from the Informed Health Choices (IHC) Key Concepts framework [16]. These were (1) an outcome may be associated with a treatment but not caused by it; (2) the results of one study considered in isolation can be misleading; (3) small studies may be misleading; (4) fair comparisons of treatments in animals or highly selected groups of people may not be relevant; and (5) beliefs alone about how treatments work are not reliable predictors of the presence or size of effects. In addition, the game was to address questions that introduced concepts of research methods and source criticism. The concepts were combined to improve critical thinking through reasoned, reflective thinking to decide what to believe and do in given situations. This is sometimes referred to as health literacy, especially critical health literacy [17]. Thus, the game will also possibly be able to increase an individual’s critical health literacy.

#### Objective

There are several reviews of game-based learning activities that encourage learning activities by building on engagement and challenges to achieve the intended learning objectives [10,11,18,19]. However, we were not able to find any studies on users’ experiences through the process from concept and development to the implementation and evaluation of a game application as a learning resource to enable people to think critically about health claims using key concepts from the IHC framework. Thus, this study aims to document how user insights and input can inform the concept and development of a game application in critical thinking about health claims for university students and collect user experiences related to the application.

## Methods

### Design and Setting

**Figure 1:**
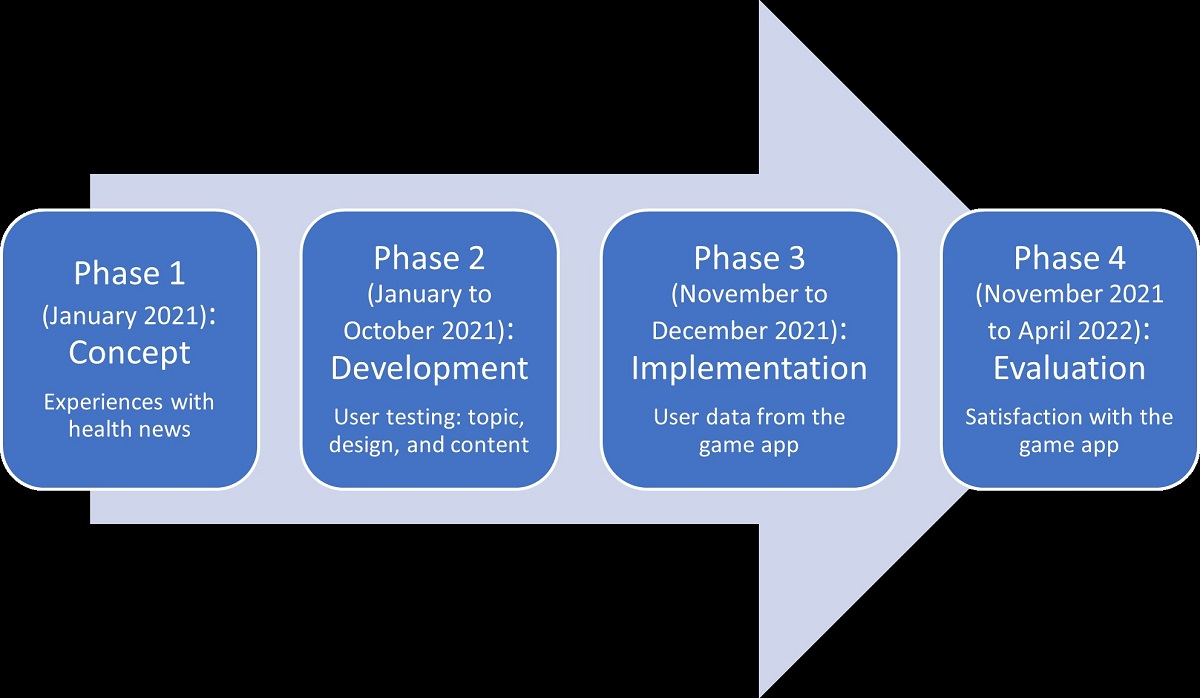
An overview of the 4 phases of the development and evaluation of the game application.

This was a mixed methods study using both quantitative and qualitative data in 4 phases (Figure 1).

The following stages are addressed:

1. Assessment of experiences with health news in unrecorded discussion (concept phase).
2. User testing of the topic, design, and content for the game application in unrecorded discussions (development phase).
3. Assessment of user patterns of the game application using user data (implementation phase).
4. Assessment of the satisfaction with the game application using a questionnaire in the game application and a recorded focus group interview (FGI; evaluation phase).

In January 2021, students’ experiences with health news in the media were used as the basis for the development of the game. After the game was developed between January 2021 and October 2021, students’ and the reference group’s experiences with a pilot of the game were used to finalize the game. The game was launched as a contest where students could win prizes from November 26 to December 14, 2021, and quantitative data from the implemented game were used to assess the uptake and use patterns of the game. Finally, students’ experiences with the implemented game were evaluated using both data from open-ended questions in the game and a recorded FGI conducted in April 2022.

### Ethical Considerations

Data in phases 1 and 2 were collected without audio recording and did not contain personal or indirect personal data. According to the guidelines of the Norwegian Centre for Research Data, approval from them was therefore not necessary. We applied for approval for the audio recording of the FGI in phase 4 from the Norwegian Centre for Research Data (reference number 910105). For all phases, we followed the research ethics guidelines of Oslo Metropolitan University (OsloMet) [20].

A reference group consisting of academics from OsloMet, students from various study programs at OsloMet, and representatives from the game developer (sikresiden.no) helped plan and develop the game application. However, the representatives from the game developer were not involved in the data analyses.

### Recruitment of Participants

Eligible participants for phase 1 were a pool of volunteer students from various Norwegian institutions of higher education who were interested in contributing to game development. Invitations to participate in an unrecorded discussion were sent to everyone in the pool between December 21, 2020, and January 25, 2021. The participants were offered a gift card of NOK 500 (US $46.9) to participate in the discussion.

In phase 2, eligible participants were a pool of volunteer students at OsloMet who were interested in participating in the research. A request for participants for unrecorded discussions was sent by MM on April 19, 2021 (Multimedia Appendix 1). The participants were offered a gift card of NOK 300 (US $28.2) to participate in the discussions.

Eligible participants for phases 3 and 4 were Norwegian-speaking students at OsloMet. In the implementation phase (phase 3) in autumn 2021, 2 research assistants were involved in marketing activities at OsloMet, for example, marketing short video clips to recruit students to try the game through the university’s social media channels (Instagram, Snapchat, and Facebook). Everyone who completed the game was entered into a lucky draw for 4 gift cards of NOK 500 (US $46.9) each, and the one with the highest score won a grand prize (wireless headphones worth NOK 5000 (US $469.4). Recruitment for recorded FGI in the evaluation phase (phase 4) took place in February and March 2022. Information about the FGI was posted on notice boards around the OsloMet campus in Oslo, and flyers were distributed to students at several campus cafeterias on 3 separate occasions. The students were offered a gift card of NOK 500 (US $46.9) to participate in the FGI.

### Data Collection

Qualitative data in phase 1 were collected through an unrecorded discussion via Zoom (Zoom Video Communications) that lasted for 1.5 hours and followed a semistructured interview guide (Multimedia Appendix 2). The purpose was to get to know the target group by exploring students’ experiences with health news in the media and how they assess the reliability of health claims. The discussion was facilitated by an employee from the developer, and MM acted as an observer who took notes.

**Figure 2:**
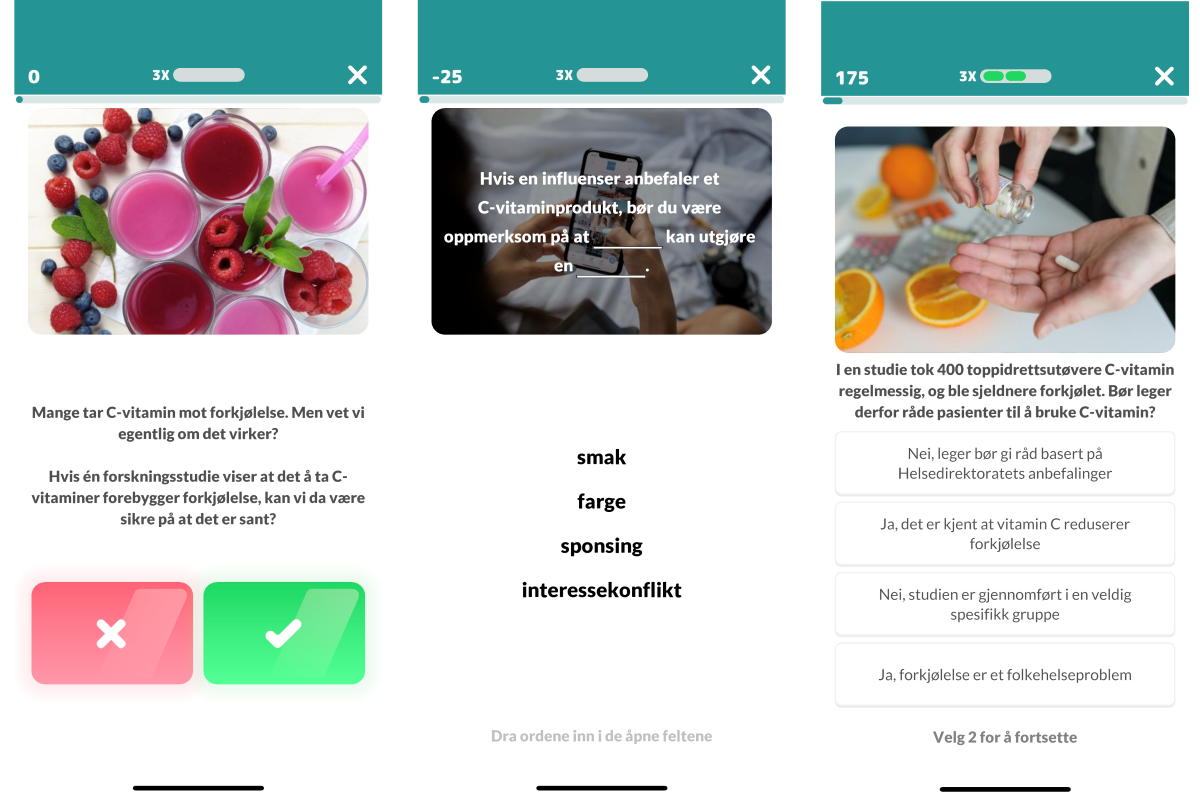
Examples of game elements (in Norwegian) assessed in phase 2.

In phase 2, qualitative data were collected through three 1.5-hour unrecorded discussions via Zoom, where the game was demonstrated question by question (see Figure 2 for examples in Norwegian and Multimedia Appendix 3 for the English translation), and the feedback was noted. The discussion was facilitated by MM, who also took notes.

Quantitative user data were collected during the implementation phase (phase 3). The data set was provided by the app developer at the request of the researchers after the competition ended in December 2021. These data constituted the quantitative data for the implementation phase (phase 3).

The game also contained a short evaluation questionnaire that appeared after the game was finished. Answering these questions was voluntary, and the questions only appeared after the first game round. The questionnaire was anonymous and administered by the app developer. The anonymous reports were handed out at the request of the researchers and constituted qualitative data for the evaluation phase (phase 4). The questionnaire consisted of 3 questions: (1) *What do you think about this way of learning?* (2) *Describe what you think was positive about the game in up to 3 words*, and (3) *Do you have any other comments or suggestions for the game?*

To collect qualitative data from the recorded FGI in phase 4, Rosenbaum’s adaption of Morville honeycomb framework for user-experience design was used to develop the interview guide [21,22] (Multimedia Appendix 4). The honeycomb framework is a tool that explains several aspects of user-experience design [21]. A 1-hour FGI via Zoom was arranged by IKOE, with LGH. as the observer, in line with the guidelines for using Zoom in research interviews at OsloMet [23]. The participants were made aware that the facilitator and observer were not involved in the development of the game. The FGI was recorded using Nettskjema, a secure solution for data collection developed and hosted by the University of Oslo [24], transcribed verbatim by IKOE, and double-checked by MM and LGH.

### Statistical Analysis

Nonparametric tests were used to analyze user data in phase 3 because the distribution was not normally distributed. Descriptive data are presented as median (range). Spearman rho was used to test the correlation between the number of replays and total points scored in the game, and the independent-samples Kruskal-Wallis test was used to test differences in points scored between faculty affiliations.

### Qualitative Data Analysis

Analysis of the unrecorded discussions in phases 1 and 2 and analysis of the open-ended questions 1 and 3 in the game survey in phase 4 was guided by a thematic analysis [25], which implied (1) gaining familiarity with the data, (2) generating codes, (3) arranging codes into subthemes, (4) reviewing subthemes, and (5) defining and naming main themes. For question (Q) 2 in the game survey, a word cloud was created using a free web-based word cloud generator (Zygomatic) [26]. Only descriptive words were extracted from the answers and used in the word cloud. Words with similar meanings were collapsed into one category (eg, “varied” and “varying” and “nice-designed” and “well-designed”). The word cloud generator also calculated the frequency of the words given in response to Q2.

For the phase 4 FGI, the findings in each facet of the honeycomb framework were summarized in tabular form and supplemented with quotes. IKOE drafted the table, whereas MM and LGH double-checked the findings against quotes. In addition, the FGI transcript was analyzed thematically [25]. The first draft was prepared by IKOE based on the analysis in NVivo (QSR International) [27] and then double-checked by MM and LGH. Disagreements were resolved by consensus among the researchers.

## Results

### Phase 1—Concept

#### Unrecorded Discussion With Students

Three students (2 women and 1 man) from different fields of study and study sites in Norway provided data on their experiences with health news. The discussion revealed that the students use a variety of media sources when reading health news: traditional newspapers (“I steal the paper when I visit my parents”), web-based newspapers (“I often read the news on my phone”), and social media (“It is much click-baits there”). When asked how they assessed whether health news is reliable or not, the students said that they had higher trust in news from the government than in the mainstream media or newspapers. They also said they had less trust in health news from commercial media (“I have less trust in paid news”).

In the discussion, the participants were shown some paid health “news” shared by influencers on Instagram and so on, and asked how they would assess their trustworthiness. They said that they were skeptical of, for example, websites that appeared to have little content that was shared by influencers or otherwise were commercial. However, it appeared that the students did not have any strategy other than “to google it” to assess whether the health news was reliable or not. One participant said, “I would not have checked the references or sources for this health news.” Two of the informants also said that they had been fooled to buy products that were aggressively advertised on social media: “You want it to work, and that is why you buy the product.” None of the participants mentioned assessing the evidence supporting the claims.

After the unrecorded discussion, MM and the representatives from the developer suggested questions for the game based on the discussion with students and the types of tasks that were technically possible. The proposed questions covered several topics and included case-based questions based on real health claims in social or other media, and the proposals were presented to the reference group.

#### Reference Group Discussion

A meeting with the reference group was held where the proposals were reviewed. The reference group decided that it would be reasonable to concentrate all questions on only one topic to make the game easier to grasp. The goal was to choose a topic that was relevant for all students regardless of their gender, age, or study specialization. The reference group concluded that the topic “dietary supplements” could fit all these requirements. The continued work was based on this idea, and a pilot of the game was created.

### Phase 2—Development

The overall impression of the game, based on 2 unrecorded discussions with students and 1 with the study’s reference group, was that it was useful, relevant, and educational; had clear, varied questions; and looked nice. The participants further expressed that learning through a game was fun, earning points was motivating, and that the competitive element was exciting. The perceived learning outcomes included both critical thinking skills and factual knowledge. Textbox 1 contains compiled feedback on the overall impression of the game.

When assessing each of the game elements, suggestions for improvements in some of the tasks included extending the time limit, minor rewording, and larger text.

**Summary of overall feedback of the game.**

Overall

- The whole game is positive—very good.
- Very cool! The questions were clear, and they were varied.
- It is fun to play, and it looks nice.

Learning through game

- Learning through games is nice.
- Liked that you had to think a bit before you answer.

Perceived learning outcomes

- How to look at an advertisement: the first thing you think: is this a scam?
- Must be more critical of what influencers say.
- Research done is not always true.
- That Vitamin C does not prevent colds.
- Learned a bit more about dietary supplements that you do not think about otherwise.

Motivational elements

- You had fun playing.
- The most fun were those that had a short time frame.
- The bubble puzzle was fun.
- You want to play more to get a higher score.
- High score ranking makes it motivating to play several times.

### Phase 3—Implementation

#### User Statistics

In total, 227 students registered in the game application during the implementation phase (phase 3). However, of these 227 students, 34 (15%) students did not test the game and were therefore excluded from the analysis, resulting in 193 (85%) students who tested the game. Of these 193 students, 70 (36.3%) students chose to answer the voluntary evaluation questions after the game.

#### App Use Data

In the game, it was possible to earn stars based on the percentage of correct answers given (1 star=1%-24%; 2 stars=25%-49%; 3 stars=50%-79%; 4 stars=80%-99%; and 5 stars=100%). All players achieved at least 3 stars, and 87% (168/193) of the students earned a minimum of 4 stars. Those achieving 3 stars (25/193, 13%) had a median replay of 1 (range 1-4), those achieving 4 stars (109/193, 56.5%) had a median replay of 1 (range 1-35), and finally, those achieving 5 stars (59/193, 30.5%) had a median replay of 5 (range 1-296; Table 1). In total, 46.6% (90/193) played the game once, whereas 4.7% (9/193) played 20 or more times (up to 296 times). The median score for all students was 4298 points, and the highest individual score in the game was 5579 points (Table 1). A correlation (*r*=0.77) was found between the number of replays and the total points achieved in the game, and the result was statistically significant (*P*<.001).

**Figure 3:**
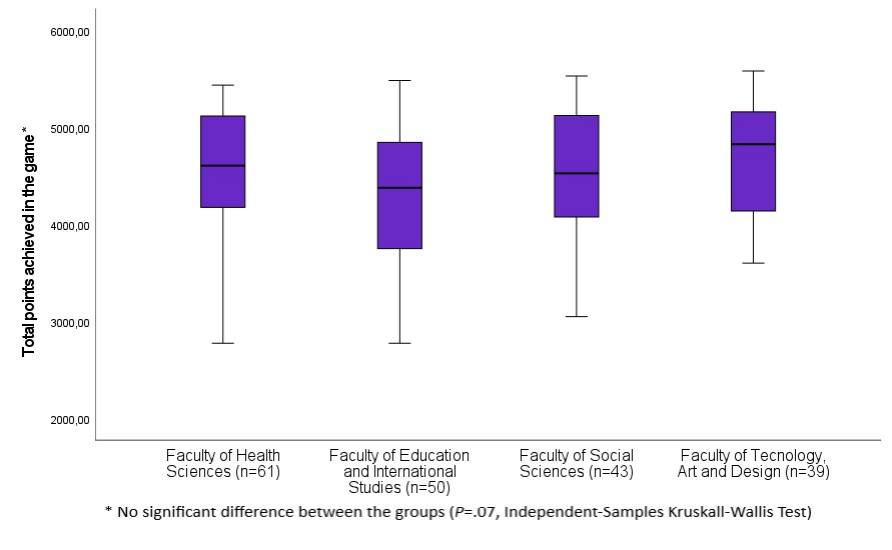
Total points achieved in the game at the different faculties.

There was no statistically significant difference (*P*=.07) between the students’ total scores from the different faculties (Figure 3).

**Table 1:** Median scores of user data.

Stars earned (% correct answers)	Values, n (%)	Number replays, median (range)	Points, median (range)
☆ (1-24)	0 (0)	0 (0)	0 (0)
☆ ☆ (25-49)	0 (0)	0 (0)	0 (0)
☆ ☆ ☆ (50-79)	25 (13)	1 (1-4)	3500 (2775-3725)
☆ ☆ ☆ ☆ (80-99)	109 (56.5)	1 (1-35)	4425 (3725-5108)
☆ ☆ ☆ ☆ ☆ (100)	59 (30.5)	5 (1-296)	5223 (5000-5579)
Total	193 (100)	2 (1-296)	4531 (2775-5579)

### Phase 4—Evaluation

#### Results From Thematic Analysis of Open-Ended Questions in the Game Application

Responses to the 2 open-ended questions “What do you think about this way of learning?” and “Do you have any other comments or suggestions for the game?” were brief. Almost half of the responses consisted of just one word, and the longest response was 2 sentences. The results from these 2 questions (Q1 and Q3) were analyzed and coded into 4 main themes: (1) “Experience of performing the game,” (2) “Experience of learning,” (3) “Feedback on the design,” and (4) “Suggestions for improvement.”

The students used words such as “fun,” “interesting,” and “educational” to describe their experiences with the game. They further used words such as “motivating,” “effective,” and “engaging” related to the learning experience. The students’ perception of the design was expressed in words such as “nice,” “user-friendly,” “colorful,” and “varied”; however, 1 participant mentioned that the font was small in some of the tasks. Suggestions for improvement varied from adding more games and modules, having available sources, and adding more difficult questions to adding a small summary about the game at the start of the game. A detailed overview of the themes and codes can be found in Multimedia Appendix 5.

The respondent wrote up to 3 words in response to the question “Describe what you think was positive about the game in up to three words” (Q2). To visualize the responses, a word cloud was generated where the words with the largest size appeared most frequently. The most frequent words used were fun (20 times), varied (11 times), informative (9 times), simple (9 times), easy (8 times), educational (6 times), and interesting (6 times; Multimedia Appendix 6).

#### Results From FGI Evaluation

Six students showed interest in participating in the recorded FGI evaluation and were provided with a link to a consent form. The participants signed the form before taking part in the FGI, but 2 participants did not, that is, they dropped out without giving a reason.

On April 8, 2022, the recorded FGI was conducted with 4 women, 2 of whom were from the Faculty of Health Sciences and 2 from the Faculty of Social Sciences. One of the participants was a master’s student, whereas the others were bachelor’s students. One participant tested the game in the introduction phase (phase 3), whereas the others had not tested the game before. All participants spoke Norwegian.

Although the data collection followed the interview guide based on Rosenbaum’s honeycomb framework (the results can be found in Multimedia Appendix 7), the key themes were as follows: (1) game design, (2) trustworthiness, (3) learning outcome, (4) target group, and (5) further development.

The informants expressed that they had experienced the game design as varied and fun with different task layouts and colors, and they liked the immediate feedback on whether the answers were correct. However, they found the design of the web-based game solution, with a mobile screen image, to be suboptimal because of small letters. On the basis of their previous knowledge of source criticism, the judgment of whether the information is likely or not, and knowledge of research, they found the game trustworthy. They were positive about using games as learning tools for critical thinking and reasoning. They further found the game useful in education, writing assignments, and in everyday life, but expressed concerns about the academic level of the game being slightly too low. They believed that the target group could be anything from university to primary school, but that there should be various task levels depending on the target group. Other suggestions for further development included having an introductory text explaining the aim of the game, learning stops along the way, and references and sources at the end. However, they still wanted to maintain a game element in the form of points. Suggestions for expanding the topic of the game included politics, climate, nutrition, physical activity, and how to read and interpret research.

## Discussion

### Principal Findings

In this study, we explored students’ experiences with assessing health news in the media as a basis for the development of a serious game to increase critical thinking about health claims and found that users’ insights and inputs can be successfully used to inform the concept and development of such a game. The students’ user patterns and evaluations of the game revealed that the users experienced the game as educational, fun, and engaging. The mixed methods design helps to better understand user experiences and makes the process replicable for those who aim to develop serious games in critical thinking about claims in other disciplines, such as climate and sustainability. In the following sections, we discuss details of the main findings of this study before a general discussion, in which we compare the results with previous studies.

The initial unrecorded discussion in our study revealed that the students were skeptical of health news shared by influencers or otherwise initiated or spread by commercial actors. Nevertheless, their assessment of whether a claim was trustworthy was limited to Google searches. The disadvantage of this strategy is that, in particular, information on treatment outcomes may be incomplete, written by nonexperts, or have commercial goals [28]. We also wanted to gain insight into whether any of the participants considered the evidence supporting the claims, and none mentioned that they did. An essential element in the development of the game was, therefore, to strengthen the students’ source criticism and build their capacity to identify the basis for claims, that is, the evidence supporting the claims about health effects through the key concepts of the IHC framework, and thus increase their critical health literacy.

In total, 87% (168/193) of participants achieved a minimum of 4 out of 5 stars, which may indicate that the degree of difficulty was somewhat low. This was supported by the findings in the open-ended questions in the game where words such as “simple” and “easy” were used, and from the FGI where one participant expressed, “I think perhaps that it was almost too easy.” A detectable correlation was found between the number of replays and total points obtained in the game, suggesting that the game may be suitable for practicing concepts. This was supported by findings from the FGI, where one participant expressed, “[…] it is good that you could learn new concepts.” It may seem that the topic of dietary supplements was perceived as generic, as there was no demonstrable difference between the total scores of students from different faculties. This was reflected in the FGI, where both students from the Faculty of Social Science and the Faculty of Health Sciences found the topic equally interesting.

Joy and higher concentration have been shown to occur when students perceive clear goals, ease of use, and usefulness during gaming [29]. The students who answered the evaluation questions in the game used words like “fun,” “interesting,” and “educational” about the experiences with the game, and words like “motivating,” “effective,” and “engaging” related to the learning experience. Furthermore, the design was described as “varied,” and “user-friendly.” The participants in the FGI described that the game was useful because it contributed to reflection, awareness-raising, and sparked interest in learning more. Critical thinking is one of the “21^st^-century skills” [30], and a 2015 review of empirical research on the use of serious games in science education highlighted the use of serious games as an educational tool to promote students’ development of these skills [31]. This was consistent with our intentions for the game, which was to increase the likelihood of believing and acting on claims that are more likely to help achieve a desired goal, that is, to increase critical thinking [32].

### Comparison With Previous Studies

Although we described user experiences with serious games, we will draw on the literature based on gamification to widen our scope, as both these terms are centered on game elements. In addition, as stated in a review of serious games for health professions, the term “serious games” is not well established; therefore, previous studies on the topic do not necessarily use this term [11].

Zainuddin et al [33] studied the role of gamified e-quizzes on student learning and engagement and found that gamified e-quiz exercises work positively to engage students in learning by involving game principles such as points, progression, competitions, certificates, and leaderboards. Similarly, we found that the points, progression, and competition made the learning experience effective and engaging. Competition, that is, earning points and an overview of the ranking, was perceived as motivating and made the students play several times, which allowed them to memorize the concepts.

Silva et al [34] claimed that gamification is not only a useful tool but can also help in the learning process. In a rapid review, Sipiyaruk et al [35] found that serious games in health care education are potentially effective learning tools in terms of improving knowledge and skills. However, the results of serious games are not consistent with those of traditional learning methods. In the evaluation of our game, the informants expressed that the game created the motivation to learn more. Therefore, we expect our game to aid the learning process of knowledge and skills by addressing intrinsic motivation, which is crucial for improving learning outcomes [13]. As a starting point, we implemented the game as an introduction to a course in evidence-based health care at OsloMet in August 2022. However, further studies are needed to determine whether games can aid the learning process and outcomes of evidence-based health care.

A different perspective in relation to serious games was used in a study by Zairi et al [36], in which medical students participated in activities to design serious games as part of the curriculum. The authors found that students learned skills related to communication, collaboration, creativity, and critical thinking when they themselves created serious games. Although this approach is not relevant to our study on game development, there may be opportunities for this in future “Behind the headlines” projects.

### Limitations

Our study has some limitations. Our first discussion in phase 1 was not audio recorded, which may raise questions of bias toward using only agenda-appropriate data. In addition, owing to the relatively low number of participants, we can discuss whether we achieved information power. Information power in qualitative research means that the more study-relevant the information the participants have, the lower the number of participants required [37]. In our case, the scope of the discussion was narrow, the participants had prior experience with health news from the media, and the interview guide was targeted. Our findings in phase 1 are also consistent with previous knowledge about how adults and upper secondary students find and assess health news in the media [38-40]. This strengthens confidence in our findings and indicates that the sample size was adequate. The unrecorded discussion in phase 2 aimed to test a pilot game and make changes before implementation. In this phase, the scope of the discussions was narrow and targeted; thus, only a few participants were needed. Phases 3 and 4 were related to the implementation and evaluation of the game, and although the number of participants in the FGI was low, we found consistency across the quantitative and qualitative data. Thus, we interpret that the number of participants in the FGI provided sufficient informational power.

Our goal with the game was to improve critical thinking among students, but it can be questioned whether the tasks in the game were sufficiently designed to develop critical thinking or whether they mainly assessed factual knowledge of the effects of dietary supplements. On the basis of our results, however, it appears that the various questions helped the students increase their awareness of the assessment of health claims in the media, and thus their critical thinking.

Finally, the study aimed to obtain user experiences from concept, development, and implementation to evaluation; thus, it was not possible to draw conclusions about the effect of the game in a learning setting. Randomized trials are necessary to assess the effects of the game.

### Reflexivity

In qualitative research, including a mixed methods approach, researchers are part of the research process, and we recognize that our previous experiences, assumptions, and beliefs influence it. MM, LGH, and LH are all professors at OsloMet and IKOE is currently a PhD candidate. The professors were lecturers at OsloMet, but IKOE had no formal relationship with any of the students at OsloMet. MM and LH contributed to the development of the game, and to avoid possible social desirability bias, that is, the tendency of research participants to answer what they believe is more socially acceptable rather than answering what reflects their true thoughts [41], IKOE and LGH, who had not participated in the development of the game, conducted the FGI. All analyses, both quantitative and qualitative, were also reviewed by several researchers to minimize the chance of misinterpretation.

### Conclusion and Practical Implications

Acting on misleading health claims can adversely affect health, and there is a need for tools and learning resources that enable people to critically assess these claims. Serious games can be developed in this regard, as they can trigger and motivate students to learn more about a subject. We found that user insights and inputs can be successfully used to inform the concept and development of a serious game that aims to engage students in critical thinking about health claims. On the basis of a mixed methods evaluation of the game, we found that users experienced the game as educational, fun, and engaging. Our results can inform the design and implementation of serious games for health educational purposes related to critical thinking about claims in other disciplines where critical thinking skills are needed, such as climate, democracy, and sustainability. Future research should focus on assessing the effects of serious games on learning outcomes and health choices in randomized trials.

## Appendix

Multimedia Appendix 1 Recruitment to participate in an unrecorded discussion, phase 2.

Multimedia Appendix 2 Interview guide for unrecorded discussion, phase 1.

Multimedia Appendix 3 Examples of game application tasks.

Multimedia Appendix 4 Interview guide for recorded focus group interviews (FGI), phase 4.

Multimedia Appendix 5 Themes and codes for Q1 and Q3, phase 4.

Multimedia Appendix 6 Frequency of words given as responses to Q2, phase 4.

Multimedia Appendix 7 Results from recorded focus group interview (FGI), phase 4.

## Abbreviations

FGI: focus group interview
IHC: Informed Health Choices
OsloMet: Oslo Metropolitan University

## Data Availability

The data sets generated and analyzed during this study are available from the corresponding author upon reasonable request. Qualitative data sets are available only in Norwegian.
