# Supplementary material for: Development and Evaluation of a Serious Game Application to Engage University Students in Critical Thinking About Health Claims: Mixed Methods Study
Source: JMIR Form Res. 2023 May 11;7:e44831. doi: 10.2196/44831 (PMC10214114; doi:10.2196/44831)
Supplement: Multimedia Appendix 1 [file formative_v7i1e44831_app1.docx]

# Multimedia Appendix 1. Recruitment to participate in an unrecorded discussion, phase 2.

A request to participate in the unrecorded discussion in Phase 2 was sent with the following requirements.

| Required information | Request |
| --- | --- |
| Name of the project and the project manager | “Behind the headlines—a game module for critical thinking”  Marianne Molin |
| Number of students | 4 participants |
| Period | Between May 1 and June 1, 2021. |
| Expected time used | 1.5 hours |
| Description of the expected contribution | We have developed a game module for critical thinking that we would like to test. The test will be carried out digitally with 2 students at a time, together with the project manager and game developers from sikresiden.no, which means that there will be two such tests of the game module. We want feedback on everything from relevance, level of difficulty for new students and language. |
| If a student with a special background/faculty/experience etc. is needed, write here: | The game will be offered to all fresh students at OsloMet (and other universities and colleges in Norway) from autumn 2021. We want all genders, from different faculties and as “fresh” as possible—preferably first-year students. |
