# Supplementary material for: Development and Evaluation of a Serious Game Application to Engage University Students in Critical Thinking About Health Claims: Mixed Methods Study
Source: JMIR Form Res. 2023 May 11;7:e44831. doi: 10.2196/44831 (PMC10214114; doi:10.2196/44831)
Supplement: Multimedia Appendix 2 [file formative_v7i1e44831_app2.docx]

# Multimedia Appendix 2. Interview guide for unrecorded discussion, phase 1.

The semi-structured interview guide (phase 1), date, and time: February 4, 2021, from 15.00 to 16.30 on Zoom.

| Questions |
| --- |
| **WHERE do you get your news from? Where do you read health news? (15 minutes)** |
| Where do you read news? Why exactly there? What makes that news more alluring than others? Are there any other sources that you use for health news? |
| Do you share health news yourself? How often do you get health news from others? What types of cases do you possibly pass on? |
| Where do you get news about COVID-19? |
|  |
| **How do you assess what you read on the web? (25 minutes)** |
| How do you know if what you read is true? What does it mean if it’s true or not? |
| Have you ever believed something in the news that turned out not to be true? |
| Now I am going to show you some examples of content from the internet/social media, and then I want you to say what you think. |
| Talk while you think! What do you think about what is written? Sender? Do you think it is easy to assess whether the claims are true or not? |
|  |
| Sponsored post from Oslo Skin Lab (screenshot from Instagram)  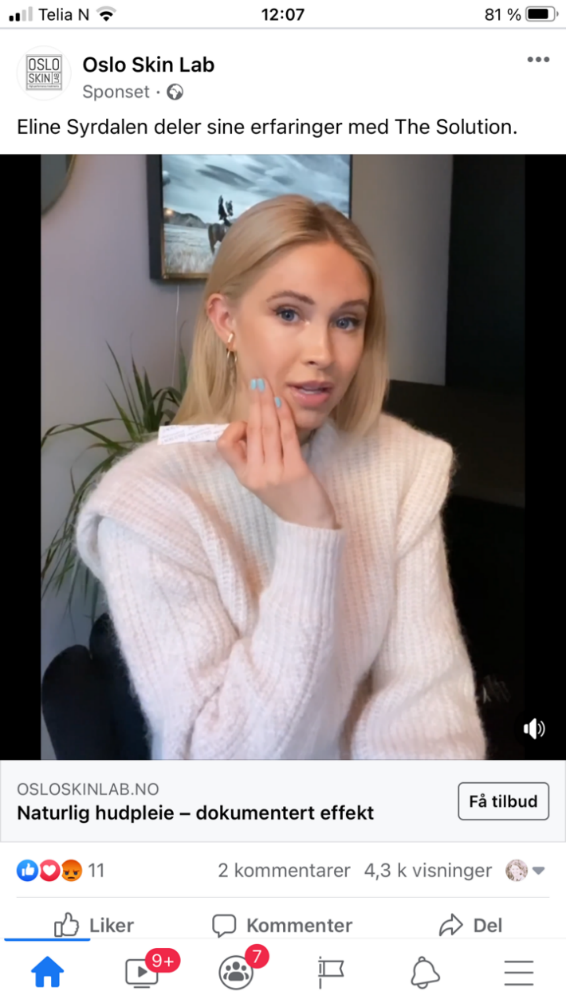  *English translation:* Eline Syrdalen shares her experiences with The Solution.  Natural skin care—documented effect. |
|  |
| Vitamin D  https://hemali.no/siste/britiske-politikere-anbefaler-na-vitamin-d-som-gratis-strakstiltak/  *English translation:* British politicians are now recommending vitamin D as a free prevention against viruses. |
|  |
| **What is “critical thinking”? Included examples (15 min)** |
| What is the first thing that comes to mind when I say, “critical thinking”? |
| How would you explain what it means in your own words?  When do you use it in your everyday life? What situations? Give examples. |
| What does it take for you to consider something untrue? What does it take for you to speak up to someone who claims something you think is not true? Name examples! |
