# Supplementary material for: Development and Evaluation of a Serious Game Application to Engage University Students in Critical Thinking About Health Claims: Mixed Methods Study
Source: JMIR Form Res. 2023 May 11;7:e44831. doi: 10.2196/44831 (PMC10214114; doi:10.2196/44831)
Supplement: Multimedia Appendix 3 [file formative_v7i1e44831_app3.docx]

# Multimedia Appendix 3. Examples of game application tasks.

The English translations of the examples shown in Figure 2 in the manuscript are presented here.


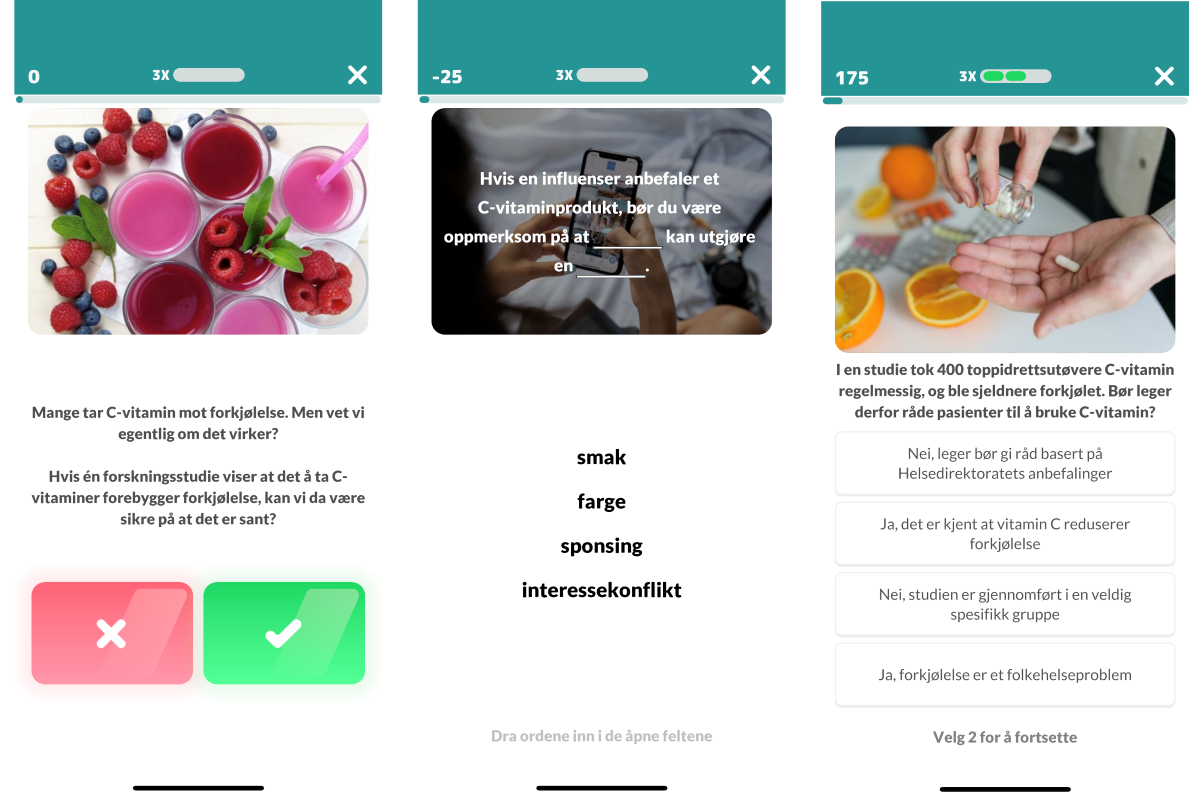


**Picture 1.** Although many people use vitamin C to prevent colds, the effectiveness of this treatment is still uncertain. Can we confidently conclude that taking vitamin C prevents colds based solely on a single research study showing such a relationship?

Answer options: No or Yes

**Picture 2**. If an influencer recommends a vitamin C product, you should be aware that __________ can constitute _____________.

Answer options (drag and drop options): taste, color, sponsorship, and conflict of interest

**Picture 3.** Given that a study showed that 400 elite athletes who regularly consumed vitamin C experienced fewer colds, is it appropriate for doctors to recommend vitamin C supplements to their patients to prevent colds?

Answer Options (Choose 2 Options)

(1) No, doctors should provide advice based on the Norwegian Directorate of Health’s recommendations.

(2) Yes, vitamin C is known to prevent the common cold.

(3) No, the study was conducted in a very specific group.

(4) Yes, the common cold is a public health concern.
