# Supplementary material for: Development and Evaluation of a Serious Game Application to Engage University Students in Critical Thinking About Health Claims: Mixed Methods Study
Source: JMIR Form Res. 2023 May 11;7:e44831. doi: 10.2196/44831 (PMC10214114; doi:10.2196/44831)
Supplement: Multimedia Appendix 4 [file formative_v7i1e44831_app4.docx]

# Multimedia Appendix 4. Interview guide for recorded focus group interviews (FGI), phase 4

The interview guide (phase 4) is based on Rosenbaum’s adaption of Merville’s honeycomb framework for user-experience design.

| Question(s) | Possible follow-up questions |
| --- | --- |
| 1. First impression  a. Do you have any immediate thoughts on anything good or bad about the game? | Ask individually one at the time |
| 2. Findability  a. How easy or difficult was it to find the necessary information and content about the game?  b. Do you have any ideas on how we can spread information about the game to relevant students? | For example, how to download the app, how to play on the web etc. |
| 3. Usefulness  a. What do you feel you are left with after testing the game?  b. How useful was what you learned in the game? | Have you learned anything?  Want to learn more about something?  Useful for, e.g.,   1. education 2. Professional practice 3. Daily life |
| 4. Understandability  a. How do you think it was to understand the content of the game?  b. How do you think it was to understand how to play the game? | Elaborate.  Difficult words/concepts?  Is it a familiar or unfamiliar field? (Dietary supplement)  Technical challenges? |
| 5. Usability  a. How easy or difficult do you think it will be to use what you have learned in the game? | Further use in, e.g.,   1. education 2. Professional practice 3. Daily life |
| 6. Credibility  a. What is your impression of the credibility of the content of the game? | (E.g., references to sources are not used in the game) |
| 7. Desirability  a. What do you think of the game?  b. What do you think of the design of the game? | How much did you like or dislike the game?  What about the design? |
| 8. Affiliation  a. Did you feel that the game was suitable for students at OsloMet regardless of their field of study?  b. Do you think this way of learning can also be suitable for other topics?  c. Do you have any ideas or suggestions for topics that could be suitable if the game were to be expanded with more modules?  d. Do you think the game will be interesting for students at other universities? | Was the topic “Dietary supplement” relevant to you (regardless of field of study, age, or gender)?  E.g., myths about sustainability/climate? |
| 9. Closing questions  a. Is there anything else you would like to say about the game that did not come up in the focus group interview?  Thank you for your participation! | Ask everyone individually.  E.g., are there suggestions for improvements of any kind? |
