# Supplementary material for: Development and Evaluation of a Serious Game Application to Engage University Students in Critical Thinking About Health Claims: Mixed Methods Study
Source: JMIR Form Res. 2023 May 11;7:e44831. doi: 10.2196/44831 (PMC10214114; doi:10.2196/44831)
Supplement: Multimedia Appendix 5 [file formative_v7i1e44831_app5.docx]

# Multimedia Appendix 5. Themes and codes for Q1 and Q3, phase 4.

Questions in the game application for use in phase 4: Q1: “What do you think about this way of learning?” Q3: “Do you have any other comments or suggestions for the game?”.

| Theme | Code |
| --- | --- |
| Experience in performing the game | Fun  Good  OK  It was fun  Nice  Interesting  Exiting  Great  Educational  Innovative  Interesting  Informative  I like it  Useful  Clear encouragement with the right answer  Easy to understand the game  Gives thoughts afterwards  Everything works. Simple. Did not take a long time  Simple not too time-consuming surprising and playful  Relevant  Addictive  Engaging low threshold points driven  Captivating thought-provoking |
| Experience of learning | Interactive  Very funny way to learn  A great way, Fun actually  A very effective way to learn.  Interactive  Motivating  Very effective  Engaging  Easier for those who struggle to keep up with long articles  You learn a lot more from active recall, and games are a good way to do this  Enjoyed the gamification part of learning related to the possibility of bonuses  Positive with points, penalties, and avatars  Positive with competition |
| Feedback on the design | Too small font in several places otherwise good  Variation in graphics  User-friendly  Nice design and easy to understand  Positive design, sound effects and colors  Creative  Systematic  Great colors  Cool design and good questions |
| Suggestions for improvements | More games and modules  Maybe a little obvious what the answer should be in a couple of places  I want to know why my answer was wrong.  Have sources available. Why is the game credible?  More difficult options  Avoidance of leading questions  Make it clearer how far in the game you have come and how much you have left  Some tasks were stressful  Sometimes a little too much text on the tasks with a time bonus  Link to where participants can read more about the problem.  Avoidance of complicated words  Maybe you could write a little summary about the team before the game starts so that you have something to build on  Make an avatar with long blond hair |
