# Supplementary material for: Development and Evaluation of a Serious Game Application to Engage University Students in Critical Thinking About Health Claims: Mixed Methods Study
Source: JMIR Form Res. 2023 May 11;7:e44831. doi: 10.2196/44831 (PMC10214114; doi:10.2196/44831)
Supplement: Multimedia Appendix 6 [file formative_v7i1e44831_app6.docx]

# Multimedia Appendix 6. Frequency of words given as responses to Q2, phase 4.

The question in the game application for use in phase 4: Q2: “Describe what you think was positive about the game in up to three words.” Word cloud and table generated from the word cloud generator ([www.wordclouds.com](http://www.wordclouds.com) by Zygomatic).


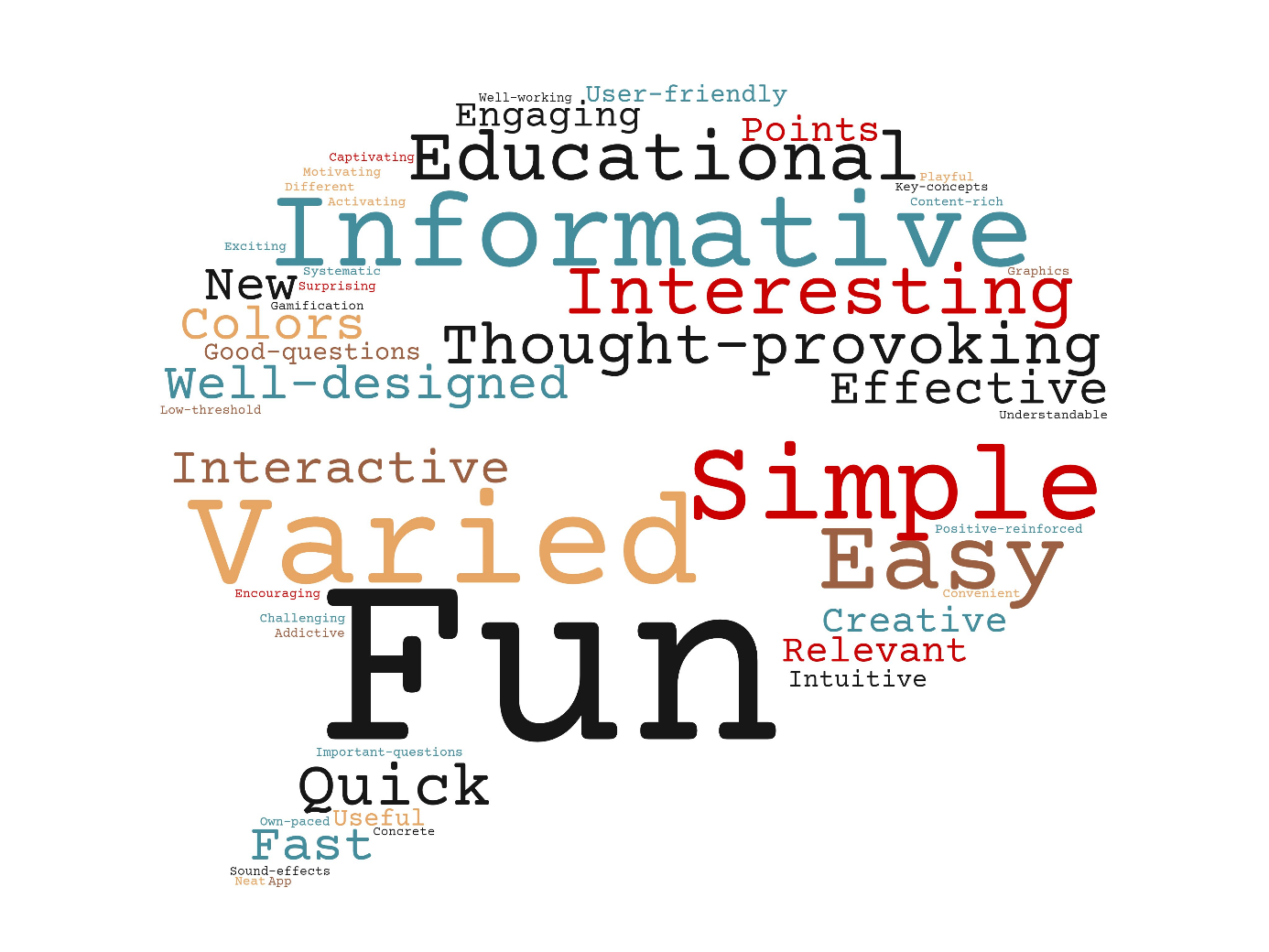


| **Word** | **Frequency** |
| --- | --- |
| Fun | 20 |
| Varied | 11 |
| Informative | 9 |
| Simple | 9 |
| Easy | 8 |
| Educational | 6 |
| Interesting | 6 |
| Quick | 5 |
| Thought-provoking | 5 |
| Colors | 4 |
| Effective | 4 |
| Fast | 4 |
| Interactive | 4 |
| New | 4 |
| Well-designed | 4 |
| Creative | 3 |
| Engaging | 3 |
| Points | 3 |
| Relevant | 3 |
| Good questions | 2 |
| Intuitive | 2 |
| Useful | 2 |
| User-friendly | 2 |
| Activating | 1 |
| Addictive | 1 |
| App | 1 |
| Captivating | 1 |
| Challenging | 1 |
| Concrete | 1 |
| Content-rich | 1 |
| Convenient | 1 |
| Different | 1 |
| Encouraging | 1 |
| Exciting | 1 |
| Gamification | 1 |
| Graphics | 1 |
| Important questions | 1 |
| Key Concepts | 1 |
| Low threshold | 1 |
| Motivating | 1 |
| Neat | 1 |
| Own-paced | 1 |
| Playful | 1 |
| Positive-reinforced | 1 |
| Sound effects | 1 |
| Surprising | 1 |
| Systematic | 1 |
| Understandable | 1 |
| Well-working | 1 |
