# Supplementary material for: Development and Evaluation of a Serious Game Application to Engage University Students in Critical Thinking About Health Claims: Mixed Methods Study
Source: JMIR Form Res. 2023 May 11;7:e44831. doi: 10.2196/44831 (PMC10214114; doi:10.2196/44831)
Supplement: Multimedia Appendix 7 [file formative_v7i1e44831_app7.docx]

# Multimedia Appendix 7. Results from recorded FGI, phase 4.

Results related to the facets of the honeycomb framework in phase 4 are presented here. The responses are directly translated from spoken Norwegian to English using Google Translate. Thus, the wording and sentence structure may be unfamiliar to an English-speaking audience.

| Facets | Findings | Quotes^a^ |
| --- | --- | --- |
| Findability | Have clear instructions for log-in | *Informant 4: now we are largely back at school, and then you can use posters with, for example, step 1 do this, step 2 write that […], step 3 do this, step 4, yes, have fun and learn.* |
|  | Posts on notice boards |  |
|  | Use of social media | *Informant 1: I also think that Instagram is often a great way to get information out, i.e., OsloMet's Instagram. Especially the stories they make [...]* |
|  | Use of competition | *Informant 2: I also think that competitions and the like make people try the game[...] if more students like it, they will recommend it to other students at UiO or UiB [...].* |
|  | Use of ambassadors |  |
|  |  |  |
| Usefulness | Contributes to reflection | *Informant 3: [...] it is good that you could learn new concepts, and[...] that you can start to reflect on the fact that you cannot trust all claims you hear.* |
|  | Awareness-raising | *Informant 2: […] we become much more aware of the choices we will make later.* |
|  | Sparks interest in learning more | *Informant 1: there were not that many tasks, so I was a bit curious to learn more, do more, actually.* |
|  | Relevant in teaching | *Informant 4: I think it might be difficult to get students to play this just for fun […]. teaching would probably have been the best, that you, yes, do it in class or something like that.* |
|  |  |  |
| Understandability | Easy to understand | *Informant 3: I think it was easy to understand, it was, and it was educational too, I think.* |
|  | Low level of difficulty | *Informant 1: I think perhaps that it was almost too easy because I know nothing about it […] but I got everything right anyway. […]. So it was a fairly easy game.* |
|  | Difficult scientific terms | *Informant 1: […] maybe more of the definitions and such, because then it was a little more difficult and you had to think a little more.* |
|  |  | *Informant 4: I am familiar with the concepts from before, but still, it was all right to get a little summary, because the concepts can easily get confusing then.* |
|  |  |  |
| Usability | Relevant for everyday life | *Informant 2:[...] I think[...] that there are things that I take with me into daily life.* |
|  | Relevant for assignment writing | *Informant 4: […]it is certainly important for all fields of study that will write assignments, use research, deliver a bachelor’s or master’s degree.* |
|  | Relevant for several subjects | *Informant 1: […] it is somewhat relevant to the journalism part, at least.* |
|  |  | *Informant 3: […] we have recently had a subject in evidence-based practice, and then a game like this could be very relevant for us in teaching.* |
|  | Regular use necessary | *Informant 3: […] you have to play it regularly to be able to use it in life going forward, I think.* |
|  |  |  |
| Credibility | Trustworthy | *Informant 2: […] the information given in this game seemed very likely.* |
|  |  | *Informant 1: I think that, for my part, the sender has quite a lot to say for credibility and the fact that I know that it is OsloMet that has made that game [...]* |
|  | Encourages information gathering | *Informant 1: […] I also feel that the game encourages a bit that you should not just believe and float on what you see, but that you should look up and check out the information yourself.* |
|  |  |  |
| Desirability | Varied and fun | *Informant 4: […] there were nice colors, it was tidy, it was funny to some extent with the different task designs.* |
|  |  | *Informant 1: I think it was really cool and made it a little more fun that there were so many different tasks then, or designs of tasks, […]* |
|  | Immediate feedback | *Informant 4: […] It was mostly fun to get the right answer and things like that. I have never come across anything like this before.* |
|  | Not optimal PC design | *Informant 3: because you have a big PC screen and then there is a small screen inside where you play, so the design right there was not very playable, I think.* |
|  |  | *Informant 1: when I played on the PC, it was like a small mobile screen on the PC, so there was some small text and something that was a little difficult to see.* |
|  |  |  |
| Affiliation | Target group | *Informant 3: it is equally relevant for all fields of study regardless of the place of study, both at the university level and even at the upper secondary level.* |
|  |  | *Informant 4: […], I think it generally contributes to critical thinking and helps to underline how important it is. […] I actually thought it could fit almost down to the secondary school level. […]. And maybe even primary school level […].* |
|  | Other relevant areas | *Informant 1: […] I thought maybe before I played that it was going to be even more about several different types of headlines […]. […] And that it could perhaps be even more interesting for most people if it was not just linked to health.* |
|  |  | *Informant 2: I think[...] politics,[...] health, maybe exercise too, eating habits, yes.* |
|  |  |  |
| Suggestions for improvement | Introductory instruction  Various levels  References | *Informant 3: I think there should be an instruction at the beginning of the game to know what to learn about.*  *I suggest […] to have different levels where you can start playing at the level that it is now, but then there will be an even more difficult and even more difficult level.*  *[…] It would have been nice to have some references at the end.* |
|  | Learning stops | *Informant 1: […] there could perhaps be even more text boxes […] that can explain a bit after you have answered questions, then maybe there could be a bit of an explanation of what is correct and why it is like that […] things appear more believable if you also get an explanation for it, not just: that’s how it is—believe in it.* |
|  | Maintain the game element | *Informant 2: I got a little more motivated to kind of keep collecting a lot of points then, and when I saw that I got the score up once more when I got faster.* |
|  |  | *Informant 1: I rather think that perhaps the competition element could go on, not time, but rather if you had been given slightly more difficult questions then the one who is able to answer the most correctly would win the competition, and that, then there is more focus on learning and you take the time to think and answer correctly and that it is not like the fastest person.* |

^a^Directly translated from spoken Norwegian to English using Google Translate — the wording and sentence structure may therefore be unfamiliar to an English-speaking audience.
